# Suppression of Mig-6 overcomes the acquired EGFR-TKI resistance of lung adenocarcinoma

**DOI:** 10.1186/s12885-020-07057-z

**Published:** 2020-06-18

**Authors:** Da Hyun Kang, Sung Soo Jung, Min-Kyung Yeo, Da Hye Lee, Geon Yoo, Sang Yeon Cho, In-Jae Oh, Ju-Ock Kim, Hee Sun Park, Chaeuk Chung, Jeong Eun Lee

**Affiliations:** 1 Division of Pulmonology, Department of Internal Medicine, College of Medicine, Chungnam National University, Daejeon, 35015 Republic of Korea; 2 Department of Pathology, College of Medicine, Chungnam National University, Daejeon, 35015 Republic of Korea; 3 Korea Institute of Toxicology, 141 Gajeong-ro, Yuseong-gu, Daejeon, 34114 Republic of Korea; 4 Chungnam National University School of Medicine, Daejeon, Republic of Korea; 5 Department of Internal Medicine, Chonnam National University Medical School, 322 Seoyangro, Hwasun-eup, Hwasun, Jeonnam 58128 Republic of Korea

**Keywords:** Mig-6, EGFR-TKI, Lung adenocarcinoma, Chemotherapy resistance

## Abstract

**Background:**

The resistance of lung cancer to epidermal growth factor receptor-tyrosine kinase inhibitor (EGFR-TKI) is one of the unconquered frontiers in chemotherapy. Mitogen-inducible gene 6 (Mig-6) is known to inhibit the kinase activity of epidermal growth factor receptor (EGFR). Similarly, numerous studies of mouse models suggested tumor suppressive function of Mig-6 in lung cancer. On the contrary, the results of clinical investigations revealed that lung cancer patients with elevated expression of Mig-6 are associated with a poor prognosis. More recent work showed that unlike wild type (WT) EGFR, mutant EGFR phosphorylates Mig-6 and phosphorylated Mig-6 negatively regulates the degradation of EGFR mutants in lung adenocarcinoma. Here, we tried to untangle the controversies surrounding Mig-6 function as a protagonist or an antagonist of EGFR-TKI resistant lung cancer.

**Methods:**

We compared the expression and phosphorylation status of Mig-6 in the EGFR-TKI resistant lung adenocarcinoma (PC9/GR cells) to EGFR-TKI sensitive lung adenocarcinoma (PC9 cells). We investigated the function of Mig-6 by either depletion or overexpression of Mig-6 in those cells and evaluated the efficacy of combining of Mig-6 knock-down and EGFR-TKI treatment in PC9/GR. The correlation between Mig-6 expressions and the prognoses of lung adenocarcinoma was examined by The Cancer Genome Atlas (TCGA) data and clinical samples.

**Results:**

Our results indicated that the expression of Mig-6 was significantly increased in PC9/GR cells compared to that of PC9 cells. The significant portion of Mig-6 existed as a phosphorylated form in PC9 and PC9/GR cells. Moreover, overexpression of Mig-6 significantly increased the cell proliferation, invasion and epithelial mesenchymal transition (EMT) in PC9 cells. Combination of Mig-6 knock-down and EGFR-TKI treatment significantly overcame the EGFR-TKI resistance of PC9/GR cells. In addition, our analyses of clinical samples confirmed that high Mig-6 expressions positively correlate with a poor prognosis and EGFR-TKI resistance in lung adenocarcinoma.

**Conclusion:**

Our findings reinforce scientific notion of Mig-6 as an oncoprotein in the context of EGFR-TKI resistant lung adenocarcinoma. We propose that targeting Mig-6 may be a promising strategy to overcome the EGFR-TKI resistance in lung cancer.

## Background

Lung cancer is still the main cause of cancer related deaths, but there have been significant improvements in the diagnosis and treatment of lung cancer over the last decade [1–6]. Targeted therapeutic agents such as epidermal growth factor receptor-tyrosine kinase inhibitors (EGFR-TKIs) and anaplastic lymphoma kinase (ALK) inhibitors, as well as immunotherapeutic drugs targeting programmed cell death protein 1 or programmed death-ligand 1 (PD-L1), have changed the treatment of lung cancer and provided other treatment options for patients with advanced and refractory lung cancer [7, 8]. Although epidermal growth factor receptor-tyrosine kinase inhibitors (EGFR-TKIs) are very effective against EGFR-mutant lung cancer, resistance to these drugs occurs within approximately 1 year, on average [9, 10]. Despite the development of third-generation EGFR-TKIs targeting T790M mutant lung cancer, resistance to EGFR-TKIs remains to be one of the most important issues affecting survival of patients. Many clinical studies are currently underway to overcome EGFR-TKI resistance [1, 3, 11]. In order to identify crucial targets of EGFR-TKI resistance, one has to initially understand regulators of EGFR signaling.

EGFR is a key regulator of cell proliferation, migration, and survival. It is one of the most frequently altered proteins in various cancers, especially in lung adenocarcinoma [12–14]. When a natural ligand binds to extracellular domain of EGFR, EGFR forms a dimer with itself and other members of the ErbB family, inducing conformational shifts that promote tyrosine autophosphorylation in the activation loop of EGFR [15, 16]. This kinase activation leads to the activation of intracellular signaling cascades, such as the PI3K/AKT, RAS/RAF/ERK, and STAT signaling pathways [17]. The EGFR signaling pathway plays an important role in the proper regulation of many metabolic, developmental, and physiological processes. Inhibition of key signal mediators downstream of EGFR may have clinical effects in the treatment of lung cancer with EGFR activity. Therefore, identifying and understanding the critical downstream effector of the oncogenic EGFR mutation may lead to the development of new therapeutic targets [18].

Mig-6 (mitogen-inducible gene 6) is known as a regulator of epidermal growth factor (EGF) signaling. It is also known as ERRFI1 (ERBB receptor feedback inhibitor 1), RALT, or gene 33, and is located at human chromosome 1p36 [19]. Mig-6 expression is induced by EGFR signaling via the RAS-MAPK pathway, as well as other mitogenic and stress stimuli. Mig-6 plays a crucial role in signal attenuation of EGFR signaling by blocking the formation of the activating dimer interface through interaction with the kinase domain of EGFR and ERBB2 [20]. Another study showed that EGFR is inhibited by the binding of Mig-6 to an activating kinase domain interface [21]. Zhang et al. reported missense and nonsense mutations in the Mig-6 coding region, as well as evidence for Mig-6 transcriptional silencing, in cancer cell lines. A study using an EGFR-mutant lung cancer mouse model showed that the loss of Mig-6 accelerates the initiation and progression of mutant EGFR-driven lung adenocarcinoma [22]. Interestingly, in vitro experimental results of same study also showed that increased tyrosine phosphorylation of Mig-6 results in decreased kinase inhibition of mutant EGFRs and block in Mig-6 mediated mutant EGFR degradation [22]. These data suggest that the interaction between Mig-6 and EGFR is very complex and vary depending on the presence or absence of EGFR mutation [22].

Contrary to the results of mouse model, the clinical manifestations reported by other groups suggested poor prognosis in patients with lung cancer with increased Mig-6 expression [20, 23]. Chang et al. demonstrated such cases in a cohort of lung cancer patients treated with gefitinib alone, where patients with a low Mig-6/EGFR ratio had higher response rates to gefitinib and markedly increased progression-free survival [20]. A similar study by Izumchenko et al. showed that a low ratio of Mig-6 to mir200 in cancer patients may serve as a promising predictive biomarker of the tumor response to EGFR-TKIs [23].

Moreover, a recently published paper showed that induction of Mig-6 under hypoxic conditions was critical for dormancy in primary cultured lung cancer cells, with activating EGFR mutations and dormant cells being more resistant to EGFR-TKIs [24]. Endo et al. also reported their analysis of the expression pattern of Mig-6 and prognosis of lung cancer patients, indicating patients with high Mig-6 expression had a poor prognosis in lung adenocarcinoma with EGFR mutation. The differences between results of animal models and clinical data suggest that the basic physiological function of Mig-6 is to inhibit EGF signaling, but it could exert an oncogenic function in certain circumstances, such as mutant EGFR or exposure to EGFR-TKI. The study of Gandhi et al. solved some of these problems by showing that contrast to WT EGFR, mutant EGFR phosphorylates Mig-6 and phosphorylation of Mig-6 negatively regulates the ubiquitination and degradation of EGFR mutants in lung adenocarcinoma. This results in sustained signaling of EGFR mutant receptor causing uncontrolled cellular proliferation and oncogenesis [25].

In this study, we tried to clarify the role of Mig-6 in lung adenocarcinoma, especially in context of EGFR-TKI-resistant lung adenocarcinoma and attempted to overcome EGFR-TKI resistance by regulating Mig-6 expression.

## Methods

### Cell lines and reagents

Human lung cancer cell lines PC9 and PC9/GR were cultured at 37 °C in 5% CO2 in RPMI-1640 medium (WELGENE, Inc., Daegu, South Korea) containing 10% fetal bovine serum (FBS; WELGENE). The EGFR-TKI gefitinib was obtained from Tocris Bioscience (Iressa, 184,475–35-2; Bristol, UK). The PC9 and PC9/GR cell lines were kindly provided by Dr. JC Lee of the Department of Oncology, University of Ulsan, Asan medical center, Republic of Korea.

### Transient transfection

Plasmids pCDNA3.1-Mig-6-FLAG and FLAG-tagged full-length Mig-6 were used for transfection. Transfections with different DNA constructs were performed using Lipofectamine LTX with Plus reagent (Invitrogen, Carlsbad, CA, USA) according to the manufacturer’s instructions. Assays were conducted after incubation for 48 h.

### Lentivirus production, infection, and establishment of stable cell lines knocking down cellular genes

Lentiviruses were prepared using human embryonic kidney 293 T cells. PC9 and PC9GR cells were infected with lentivirus to knock down cellular human Mig-6 genes. The expression arrest pLKO lentiviral vector encoding a non-silencing control short hairpin RNA (shRNA) or Mig-6 shRNA was obtained from Sigma-Aldrich (St. Louis, MO, USA). Virus supernatant plus 2 ng/mL polybrene was applied to 70–80% confluent cells, and non-infected cells were eliminated though puromycin selection.

### Western blot analysis

Cells were harvested and suspended in protein lysis buffer (Translab, Korea) and heated at 100 °C for 10 min. Protein concentration was determined using the Bio-Rad protein assay (Bio-Rad Laboratories, Hercules, CA, USA; cat. no. 500–0006). Approximately 30 μg of protein was separated by 10% sodium dodecyl sulfate polyacrylamide gel electrophoresis (SDS-PAGE) and then transferred to a polyvinylidene difluoride (PVDF) membrane (Millipore, Darmstadt, Germany). The following antibodies were used: anti-β-actin (sc-47,778, Santa Cruz Biotechnology, Dallas, TX, USA), anti-Mig-6 (GTX116560, GeneTex, Irvine, CA, USA), anti-p-EGFR (Tyr1068) (#2236, Cell Signaling Technology, Danvers, MA, USA), anti-p-EGFR (Tyr1045) (#2237, Cell Signaling Technology), anti-EGFR (sc-03, Santa Cruz Biotechnology), anti-p-AKT (#40605, Cell Signaling Technology), anti-AKT (sc-1619, Santa Cruz Biotechnology), anti-p-ERK (sc-7383, Santa Cruz Biotechnology), anti-ERK (#9102, Cell Signaling Technology), anti-E-cadherin (610,182, BD Biosciences, San Jose, CA, USA), anti-ZO1 (ab59720, Abcam, Cambridge, UK), anti-vimentin (550,513, BD Biosciences), C-MET (#3148, Cell signal) and anti-PARP (#9542, Cell Signaling Technology). Blots were developed using an enhanced chemiluminescence detection kit (Thermo Fisher Scientific, Waltham, MA, USA).

### Phos-tag immunoblot analysis

To check the phosphorylated status of Mig-6 in the lysates, we employed phos-tag SDS-PAGE. The lysate was loaded in 6% Acrylamide 100uM Mn2 + −Phos-tag™ Acrylamide. To confirm that those slower migrating bands are really phosphorylated species of Mig-6, we prepared the dephosphorylated sample by treating a part of the lysate with protein phosphatase for 9 h.

### Clinical specimens

Twenty-six pairs of lung tissue sections were cut from paraffin blocks. Tumor tissues were obtained from Chungnam National University Hospital and Chonnam National University Hospital. All methods were carried out in accordance with relevant guidelines and regulations.

Written informed consent was obtained from all patients and the study protocol was approved by the Clinical Research Ethics Committee of Chungnam National University Hospital. Institutional review board (IRB) approved this research. The IRB file number assigned to the study was 2015–07–001-002. All experiments were performed in accordance with relevant guidelines and regulations.

### Immunohistochemistry staining analysis

Tissue sections were mounted on the coated slides, deparaffinized with xylene, hydrated in serial solutions of alcohol, and heated in a pressure cooker containing 10 mmol/L sodium citrate (pH 6.0) for 3 min at full power for antigen retrieval. Endogenous peroxidase activity blocking was performed using 0.03% hydrogen peroxide containing sodium azide for 5 min. The sections were incubated at room temperature for 4 h with the rabbit polycloncal anti-ERRFI1 (Mig-6) antibody (1,50, ab198834, Abcam, Cambridge, United Kingdom). After washing, the samples were incubated in labelled polymer-HRP anti-mouse (Dako EnVision+system-HRP (DAB), Dako, Carpinteria, California, USA) for an additional 20 min at room temperature followed by additional washing. After rinsing, chromogen was developed for 2 min. The slides were then counterstained with Meyer’s hematoxylin, dehydrated, and coverslipped. Immunohistochemistry staining was scored to evaluate both intensity of the staining and proportion of stained tumor cells in each stained slide. The intensity was scored as 0 (negative), + 1 (mild), + 2 (moderate), + 3 (marked) and proportions were scored ranged from 0 to 100%.

### Immunofluorescence

Cultured cells were fixed with 4% paraformaldehyde at room temperature, permeabilized with 0.1% Triton X-100 in phosphate-buffered saline (PBS) and blocked with 3% FBS in PBS. Following overnight incubation at 4 °C with the appropriate primary antibody and incubation in the dark with Alexa 594 Fluor dye-labeled secondary antibodies, immunofluorescence was detected using a fluorescence microscope (OLYMPUS, Tokyo, Japan).

### Transwell migration and invasion assays

Migration and invasion assays were performed using a transwell membrane (0.8 μm pore-size cell culture insert, FALCON, Abilene, TX, USA). A non-coated transwell membrane was used for the cell migration assay, and a transwell membrane coated with 2 mg/mL Matrigel was used for the cell invasion assay. A total of 2 × 10^4^ cells were used for the migration assays. For the invasion assays, 2 × 10^5^ cells in serum-free medium were seeded onto the upper chamber and 750 μL medium supplemented with 10% FBS was added to the lower chamber. After incubation for 16 h, cells that did not migrate through or invade the pores were removed with a cotton swab. Cells that exhibited migration and invasion (adhered to the lower surface) were fixed with 4% paraformaldehyde, stained with 0.1% crystal violet and counted using a light microscope in three randomly selected fields. The experiments were performed thrice in triplicate.

### Scratch-wound healing and cell viability assays

Cells were seeded onto 6-well plates to 80–90% confluence and the cell monolayer was scratched in a straight line using a 200 μL pipette tip. Images were taken at 0 and 24 h after the scratch to calculate the cell migration rate.

Cell viability was counted using the CCK-8 assay kit (Dojindo Molecular Technologies, Inc., Rockville, MD, USA) following the manufacturer’s instructions. All experiments were performed three times in triplicate.

### Reverse transcription polymerase chain reaction (RT-PCR)

Cells were collected for RNA extraction. Total RNA was isolated using TRIzol reagent (Invitrogen) following the manufacturer’s instructions. cDNA was synthesized using oligo (dT) primers. The primers used for PCR amplification were as follows: hMig-6 (sense 5′-TGC ATT CTG CCC ATT ATT GA − 3′ and antisense 5′-AGG TAT GGT GGT CGT TCA GG − 3′), hE-cadherin (sense 5′-GAA CTC AGC CAA GTG TAA AAG CC − 3′ and antisense 5′-GAG TCT GAA CTG ACT TCC GC − 3′), hVimentin (sense 5′-AAA GTG CTG CCA AGA AC − 3′ and antisense 5′-AGC CTC AGA GAG GTC AGC AA − 3′), and hGAPDH (sense 5′- ACC ACA GTC CAT GCC ATC AC − 3′ and antisense 5′- TCC ACC ACC CTG TTG CTG T -3′). PCR products were electrophoresed on a 1% agarose gel and visualized by ethidium bromide staining.

### TUNEL assay

The terminal deoxynucleotidyl transferase (TdT)-mediated dUTP nick end-labeling (TUNEL) assay was performed in cells using an In Situ Cell Death Detection Kit, TMR red (Roche, Basel, Switzerland) according to the manufacturer’s protocol. Nuclei were visualized using 4′,6-diamidino-2-phenylindole (DAPI). TUNEL-positive (red) and DAPI-positive (blue) staining patterns were acquired with a fluorescence microscope (OLYMPUS) in three randomly selected fields. The experiments were performed thrice in triplicate.

### Genomic and clinical data sets

All genomic data of lung adenocarcinoma were obtained from The Cancer Genome Atlas (TCGA) data portal (https://tcga-data.nci.nih.gov) and cancer browser (https://genome-cancer.ucsc.edu). Clinical data in patients with lung adenocarcinoma (*n* = 514), gene expression data from mRNA-seq, and protein expression data from Reverse Phase Protein Array (RPPA) data were analyzed. Clinical parameters included age, gender, smoking history, TNM stage, and EGFR mutation state.

### Selection of specific gene signatures and functional enrichment analysis in relation to ERRFI1 expression

To investigate the role of Mig-6 in lung adenocarcinoma, patients were divided into two groups according to ERRFI1 expression. Based on the median value, 257 patients were included in the high ERRFI1 expression group and the low ERRFI1 expression group and Gene Ontology Consortium (GOC) was utilized to select specific gene signatures. Genes associated with the canonical pathway in the Gene Ontology (GO) database were analyzed. Patients with EGFR mutations were also analyzed separately. Gene Set Enrichment Analysis (GSEA) was utilized to enrich the mRNAs predicted to correlate with pathways in the hallmark and the Kyoto Encyclopedia of Genes and Genomes (KEGG) database.

### Survival analysis

Survival data of lung adenocarcinoma from TCGA project were obtained from TCGA data portal and cancer browser. Overall survival (OS) was measured from the date of diagnosis to the date of death or last follow-up. Survival was estimated using the Kaplan-Meier method, and survival rates were compared using the log-rank test.

### Statistical analysis

The clinical pathological data were compared using the chi-square test and the paired t-test. Patients were divided into two groups according to ERRFI1 expression as described above. In order to select differentially expressed genes between the two groups, false discovery rate-adjusted *P*-values (< 0.05) were used to correct for the Benjamini-Hochberg method. All in vitro experiments were repeated three times, and statistical significance was analyzed using a two-tailed student’s t-test or one-way analysis of variance followed by Tukey’s post hoc test. A *P*-value < 0.05 was considered statistically significant (*P < 0.05, **P < 0.01). SPSS software (version 20; IBM Corp., Armonk, NY, USA) was used for all statistical analyses.

## Results

### Mig-6 expression is higher in Gefitinib-resistant PC9 cells than in parental PC9 cells

**Fig. 1:**
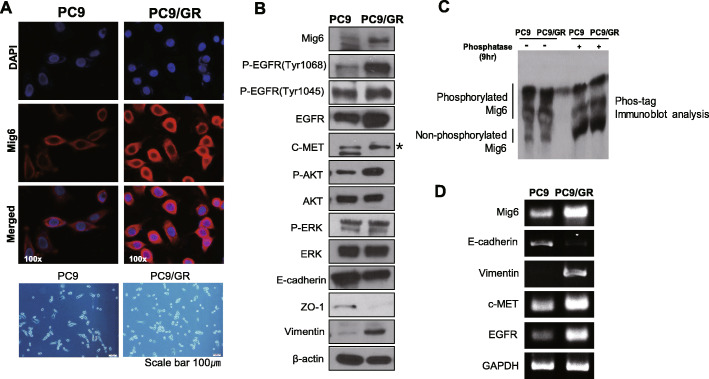
Mig-6 is overexpressed in gefitinib-resistant PC9/GR cell lines compared with parental PC9 cell lines. **a** Mig-6 expression was examined by immunofluorescence staining of endogenous Mig-6 (red) in parental PC9 cells and in PC9/GR cells (Original magnification, × 100 as displayed in the figures), lower panel is optical microscopy images of PC9 and PC9/GR cells (scale bar represents 100 μm). **b** Western blot experiments for proteins related to epidermal growth factor receptor (EGFR) signaling and the epithelial-mesenchymal transition (EMT) in PC9 and PC9/GR cells. The expressions Mig6, p-EGFR (Tyr1068), p-EGFR (Tyr1045), EGFR, c-MET, p-AKT, AKT, p-ERK, ERK, E-cadherin, Zo-1 and Vimentin were evaluated. β-actin was used as a loading control. **c** Phos-tag immunoblot analysis of Mig-6 with or without phosphatase. To check the phosphorylated status of Mig-6, the lysates we employed phos-tag SDS-PAGE. The lysate was loaded in 6% Acrylamide 100uM Mn^2+^-Phos-tag™ Acrylamide. To confirm that those slower migrating bands are really phosphorylated species of Mig-6, we treated a part of the lysate with protein phosphatase for 9 h. **d** RT-PCR analysis. mRNA levels of EGFR, E-cadherin, Vimentin, and c-MET was evaluated in PC9 and PC9/GR. GAPDH was used as an internal control. Uncropped blots were shown in Additional file 1: Fig. S1

We examined Mig-6 expression in PC9 cells harboring the EGFR exon 19 deletion and PC9/GR cells, which have EGFR-TKI resistance with an acquired T790M mutation. Western blotting and immunofluorescence analyses showed that Mig-6 expression was significantly increased in PC9/GR cells (Fig. 1a and b, Additional file 1: Fig. S1). Consistent with a previous paper [26], PC9/GR cells showed epithelial-mesenchymal transition (EMT) characteristics, including decreased E-cadherin and increased Vimentin expression (Fig. 1b). Interestingly, our data demonstrated that PC9/GR cells had significantly higher expression of EGFR, phosphorylated EGFR and AKT. The experiment using phos-tag SDS-PAGE indicated that most of Mig-6 was phosphorylated in PC9 and PC9/GR. To clarify that those slower migrating bands were really phosphorylated Mig-6, we treated the lysates with protein phosphatase for 9 h (Fig. 1c, Additional file 1: Fig. S1).

These data are consistent with the previous study showing that mutant EGFR phosphorylates Mig-6 and phosphorylation of Mig-6 negatively regulates the ubiquitination and degradation of EGFR mutants in lung adenocarcinoma [25]. PC9/GR cell showed high expression of c-MET which is one of hallmarks of the EGFR-TKI-resistant mechanism. To determine whether Mig-6 is involved in EGFR transcription, the mRNA levels were examined. Notably, the mRNA level of EGFR was significantly higher in PC9/GR cells (Fig. 1d). These results suggest that Mig-6 may be involved in EGFR-TKI resistance and the regulation of EGFR expression.

### Overexpression of Mig-6 increased cell proliferation and invasion and imparted EGFR-TKI resistance in PC9 cells

**Fig. 2:**
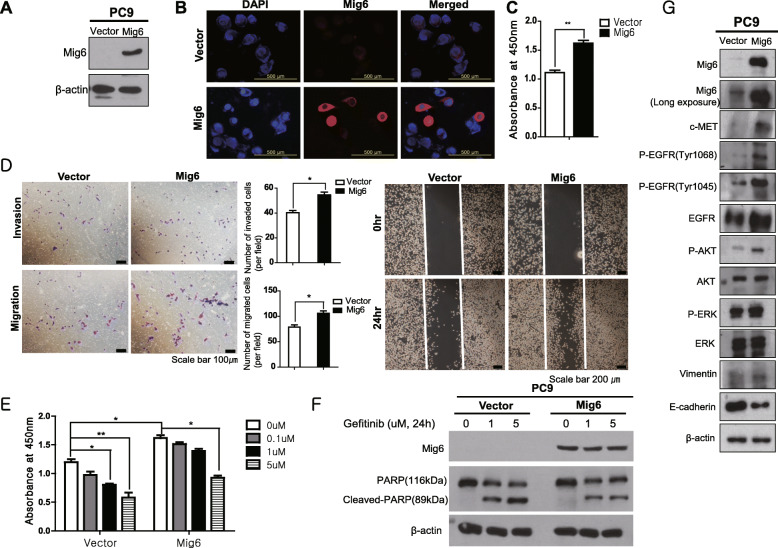
Mig-6 overexpression increased the viability and migration of PC9 cells. **a** and **b** Mig-6 expression was examined by western blot and immunofluorescence staining of endogenous Mig-6 in PC9 cells and Mig-6-overexpressing PC9 cells. **c** The cell viability of PC9 and Mig-6-overexpressing PC9 cells was measured using the Cell Counting Kit-8 (CCK-8) assay. The optical density values were detected on a microplate reader at 450 nm. **d** Cell migration ability was tested using wound-healing and transwell migration assays. Boyden chambers were stained with crystal violet for the transwell migration and invasion assays. The number of cells was counted under a microscope (*P < 0.05, **P < 0.01). **e** The viability of cells treated with different EGFR-TKI doses was measured using the CCK-8 assay. **f** PC9 cells and Mig-6-overexpressing PC9 cells were incubated with medium alone (control) or various concentrations of gefitinib for 24 h, and cleaved PARP/PARP expression was assessed by western blot analysis. **g** Proteins related to epidermal growth factor receptor (EGFR) signaling were checked with Western blot in PC9 and Mig-6 overexpressed PC9 cells. The expressions Mig6, p-EGFR (Tyr1068), p-EGFR (Tyr1045), EGFR, C-MET, p-AKT, AKT, p-ERK, ERK, E-cadherin, Zo-1 and Vimentin were evaluated. β-actin was used as a loading control. Uncropped blots were shown in Additional file 1: Fig. S2

To evaluate the effect of Mig-6 on cell proliferation, invasion, and EGFR-TKI resistance, we compared the Mig-6 overexpressed PC9 cells and control PC9 cells (Fig. 2a and b, Additional file 1: Fig. S2). The CCK-8 cell proliferation assay and migration and invasion assays were conducted. Mig-6 overexpression significantly increased PC9 cell proliferation, migration, and invasion (Fig. 2c and d). Wound healing assay also revealed that Mig-6 overexpressed PC9 cells displayed much enhanced ability to close a wound compared to control PC9 cells (Fig. 2d). PC9 cells were treated with various concentrations of the EGFR-TKI and cell viability and apoptosis were evaluated. Mig-6-overexpressing PC9 cells were more resistant to the EGFR-TKI, and cleaved PARP was significantly decreased in Mig-6-overexpressing PC9 cells compared to PC9 cells (Fig. 2e and f, Additional file 1: Fig. S2). These data suggest that Mig-6 exerts an oncogenic function in the contexts such as EGFR mutant lung cancer and exposure to EGFR-TKI.

To uncover the mechanism of these changes, we examined the expression of important signaling proteins. Mig-6-overexpressing PC9 cells showed significantly higher expression of c-MET, EGFR, p-EGFR, and p-AKT, and lower E-cadherin expression compared to PC9 cells (Fig. 2g, Additional file 1: Fig. S2). These data suggest that Mig-6 is involved in the regulation of EGFR expression and activation and induces EMT of PC9 cells.

### Knockdown of Mig-6 inhibited cell proliferation and invasion in PC9/GR cells and reversed EMT phenotype of PC9/GR cells

**Fig. 3:**
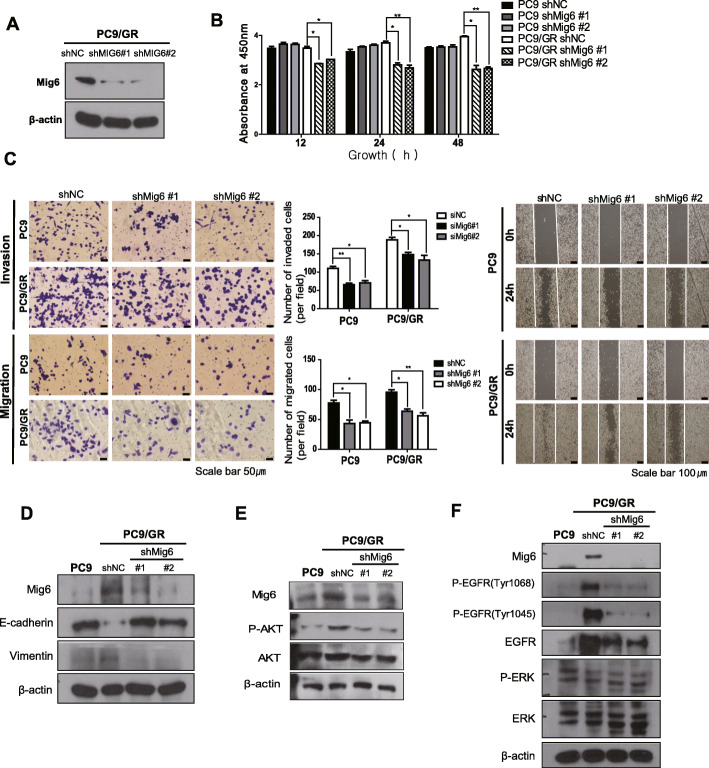
Mig-6 regulates the proliferation and migration of lung adenocarcinoma cells. **a** Western blot analysis of Mig-6 in PC/GR cells following transfection with Mig-6 siRNA or scrambled siRNA. **b** Cell viability was assessed using the CCK-8 assay after transfection of PC9 and PC9/GR cells with Mig-6 siRNA or scrambled siRNA. **c** Cell migration ability was tested using wound-healing and transwell migration assays. PC9 and PC9/GR cells were transfected with vector or siMig-6. Movement of cells into wounds is shown at 0 and 24 h post-scratching. Scale bar: 200 mm. Data are shown as the mean ± standard deviation (SD); *P < 0.05, **P < 0.01, ***P < 0.001, Student’s *t*-test. **d** Western blot analysis of E-cadherin and Vimentin in PC9 and PC9/GR cells with Mig-6 siRNA or scrambled siRNA. **e** Western blot analysis of AKT and p-AKT in PC9 and PC9/GR cells with Mig-6 siRNA or scrambled siRNA. **f** Western blot analysis of EGFR, p-EGFR (Tyr1068), p-EGFR (Tyr1045), ERK and p-ERK in PC9 and PC9/GR cells with Mig-6 siRNA or scrambled siRNA. β-actin was used as a loading control. Uncropped blots were shown in Additional file 1: Fig. S3

Next, Mig-6 shRNA PC9/GR stable cell lines were generated by transfection with a lentivirus (Fig. 3a, Additional file 1: Fig. S3). Mig-6 knockdown significantly inhibited cell proliferation in both PC9 and PC9/GR cells (Fig. 3b). Mig-6 knockdown also attenuated the cell migration and invasion of PC9 and PC9/GR cells (Fig. 3c). Wound healing assay revealed that Mig-6 shRNA PC9/GR cells displayed much decreased the ability of cells to close a wound compared to control PC9/GR cells (Fig. 3c). Knockdown of Mig-6 in PC9/GR cells decreased the expression of Vimentin and increased that of E-cadherin, suggesting loss of mesenchymal phenotype upon inhibiting Mig-6 (Fig. 3d, Additional file 1: Fig. S3). Mig-6 shRNA PC9/GR cells demonstrated significantly lower expression of EGFR, p-EGFR, p-ERK and p-AKT compared to control PC9/GR cells (Fig. 3e and f, Additional file 1: Fig. S3). These data are content with recent study’s results showing that phosphorylated Mig-6 negatively inhibits degradation of EGFR mutants in lung adenocarcinoma and causes sustained signaling of EGFR signaling causing uncontrolled cellular proliferation and oncogenesis [25].

### Knockdown of Mig-6 and treatment with an EGFR-TKI has a synergistic effect in killing EGFR-TKI-resistant adenocarcinoma

**Fig. 4:**
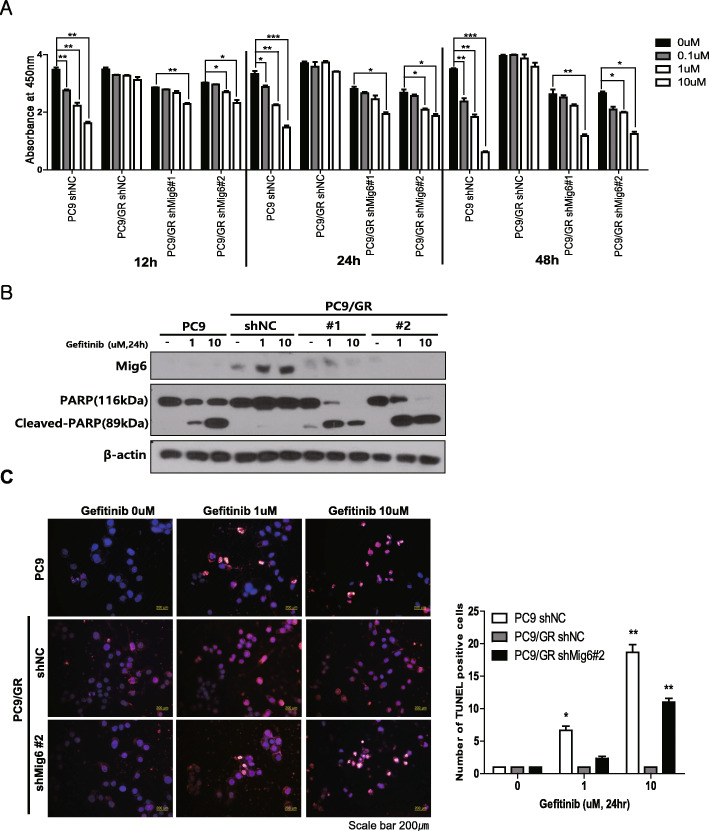
Mig-6 inhibition restores sensitivity to EGFR-tyrosine kinase inhibitors (TKIs) in PC9/GR cells. **a** Cell viability was assessed using the CCK-8 assay after transfection with Mig-6 siRNA or scrambled siRNA and treatment with various doses of gefitinib. Data are shown as the mean ± SD; *P < 0.05, **P < 0.01, ***P < 0.001, Student’s *t*-test. **b** PC9 and PC9/GR cells were incubated with medium alone (control) or various concentrations of gefitinib for 24 h with or without siMig-6, and cleaved PARP/PARP expression was assessed by western blot analysis. β-actin was used as a loading control. **c** Apoptotic cells were evaluated by TUNEL assays. The number of apoptotic cells (TUNEL-stained) and the total number of cells (DAPI-stained) were counted to determine the percentage of apoptotic cells in the tumor sections. An apparent increase in the number of apoptotic PC9/GR cells was observed when cells were simultaneously treated with the EGFR-TKI and siMig-6. Data are shown as the mean ± SD; *P < 0.05, **P < 0.01, ***P < 0.001, Student’s *t*-test. Uncropped blots were shown in Additional file 1: Fig. S4

To determine whether knockdown of Mig-6 and treatment with an EGFR-TKI has a synergistic effect in killing EGFR-TKI-resistant lung adenocarcinoma, we knocked down Mig-6 using shRNA and evaluated the effect on cell proliferation and EGFR-TKI sensitivity. Mig-6 knockdown alone slightly decreased the proliferation of PC9 and PC9/GR cells. Treatment with the EGFR-TKI alone did not affect the viability PC9/GR cells. However, the combination of Mig-6 knockdown and EGFR-TKI treatment markedly decreased the viability of PC9/GR cells (Fig. 4a). Consistent with these data, the level of PARP cleavage was higher when PC9/GR cells were simultaneously treated with Mig-6 knock down and the EGFR-TKI (Fig. 4b, Additional file 1: Fig. S4). The TUNEL assay also showed a markedly higher level of apoptosis upon the combination of Mig-6 knock down and the EGFR-TKI treatment (Fig. 4c).

Interestingly, EGFR-TKI treatment significantly increased the expression of Mig-6 in PC9/GR cells (Fig. 4b, Additional file 1: Fig. S4). This result suggests a crucial role for Mig-6 in cell survival and EGFR-TKI resistance, especially when PC9/GR cells are exposed to EGFR-TKIs. Therefore, targeting Mig-6 with EGFR-TKIs may be an effective strategy for killing EGFR-TKI-resistant lung adenocarcinoma.

### High Mig-6 expression is related to poor prognosis of lung adenocarcinoma and EMT signaling

**Fig. 5:**
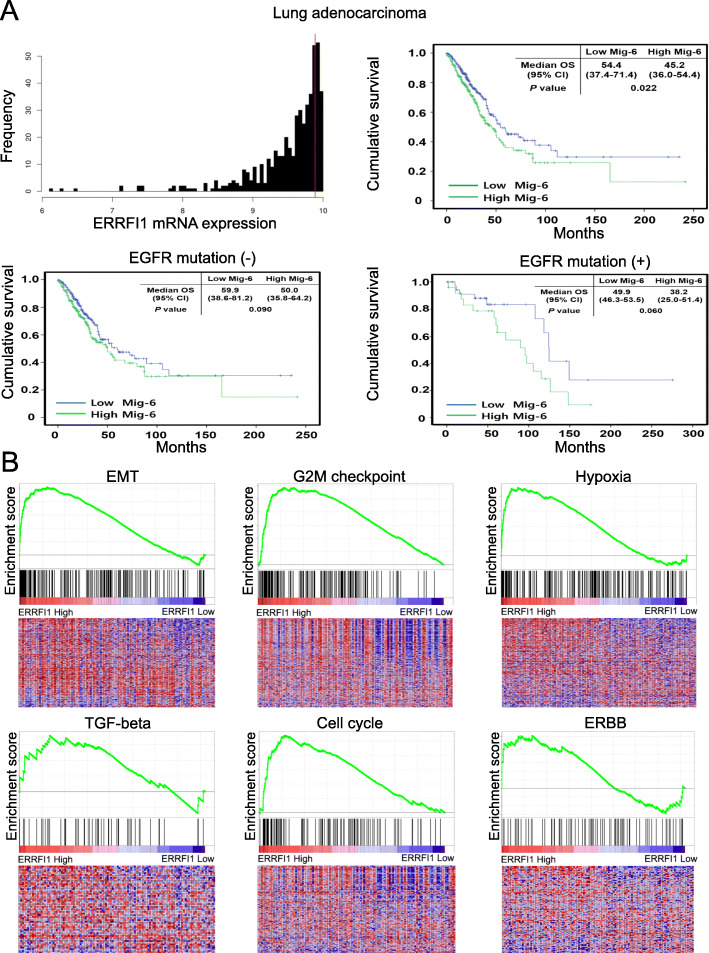
High expression of Mig-6 is correlated with poor prognosis and EMT in lung cancer. **a** Overall survival of lung adenocarcinoma patients was compared between Mig-6-high and Mig-6-low expression groups. Histograms of Mig-6 gene expression in lung adenocarcinoma patients. Vertical red lines designate 9.887 as the optimal cutoff of mRNA derived from the log-rank test. High mRNA expression of Mig-6 was significantly associated with a poor prognosis (hazard ratio [HR]: 1.92 [95% confidence interval (CI): 1.29–2.85]). Subgroup analysis was performed in patients with or without EGFR mutation. High mRNA expression of Mig-6 without EGFR mutation was significantly associated with poor prognosis (HR: 1.86 [95% CI: 1.19–2.9]). High mRNA expression of Mig-6 with EGFR mutation was also significantly associated with poor prognosis (HR: 2.33 [95% CI: 0.97–5.59]). **b** The graph displays the enrichment gene set based on the hallmark and Kyoto Encyclopedia of Genes and Genomes (KEGG) pathways in the top 10% vs. bottom 10% groups of Mig-6 in lung adenocarcinoma patients. Gene Set Enrichment Analysis of the EMT, G2M checkpoint, hypoxia, TGF-β, cell cycle, and ERBB signature genes in the high and low Mig-6 expression groups of lung adenocarcinoma patients

Next, we analyzed whether Mig-6 expression correlated with the prognosis of lung adenocarcinoma using TCGA data. RNA sequencing data of 514 lung adenocarcinoma patients were classified as Mig-6 high or low and then the OS of patients was analyzed (Table S1). Patients with high Mig-6 expression had a poor prognosis (Fig. 5a). To identify the effect of EGFR mutation on survival, a subgroup analysis was performed. In both groups (with and without EGFR mutation), patients with high Mig-6 expression had a poor prognosis. Patients with high Mig-6 expression also exhibited significantly more aggressive phenotypes, including high T stage and lymph node metastasis, than those with low Mig-6 expression (Table S2).

In addition, GSEA was used to identify pathway enrichments related to Mig-6. Some signature genes related to EMT, G2M checkpoint, hypoxia, TGF-β, cell cycle, and ERBB were significantly increased in the high Mig-6 expression group (Fig. 5b). These results reveal that Mig-6 plays a crucial function in the tumor progression and EMT of lung adenocarcinoma.

GO analysis revealed that the lung adenocarcinoma patients with high Mig-6 expression have upregulation of genes related to the mitotic cell cycle, positive regulation of cell proliferation, angiogenesis, positive regulation of cell migration, response to hypoxia, and positive regulation of cell differentiation (Table S3). We performed the same analysis separately in lung adenocarcinoma patients with EGFR mutation. In the subgroup with high Mig-6 expression and EGFR mutation, genes involved in canonical glycolysis, inflammatory response, cytokine-mediated signaling pathway, and positive response to tumor necrosis factor were also elevated (Table S4).

### Analysis of Mig-6 expression in lung adenocarcinoma between the initial biopsy and after acquiring EGFR-TKI resistance

**Fig. 6:**
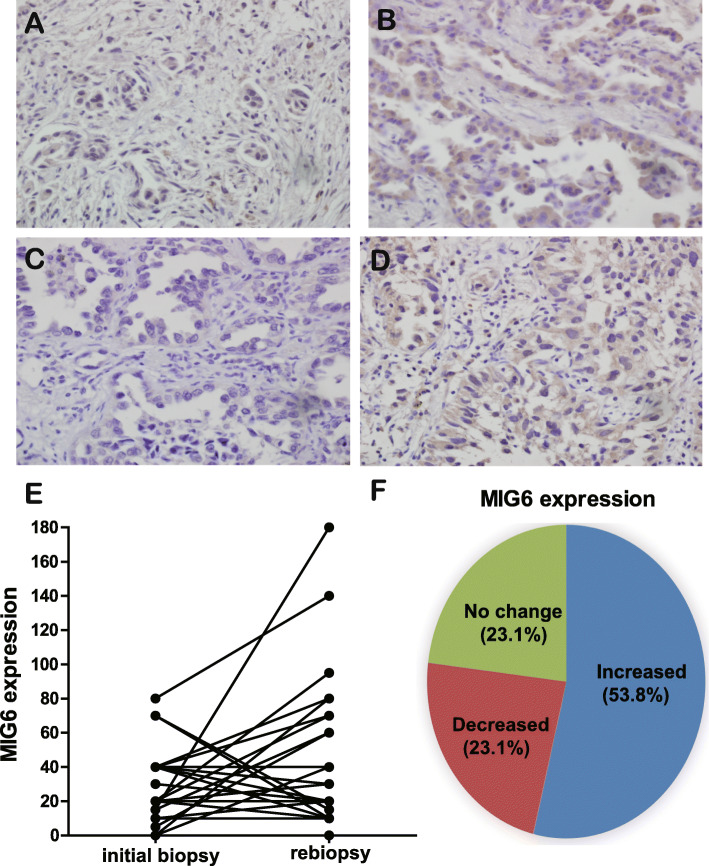
Comparison of Mig-6 expression in lung adenocarcinoma at baseline and after acquiring EGFR-TKI resistance. **a–d** Representative pictures of Mig-6 by immunohistochemistry in cases 1 and 2. **a** and **c** are initial biopsy samples. **b** and **d** are matched re-biopsy samples of case 1 and 2. The expression of Mig-6 in re-biopsy samples were significantly higher than those of initial biopsy samples. **e** and **f** Analysis of Mig-6 expressions at baseline and after acquiring EGFR-TKI resistance (*n* = 26)

To validate the clinical relevance of this study, we compared the expression of Mig-6 in lung adenocarcinoma specimens obtained from patients both before treatment with EGFR-TKIs and after the development of acquired EGFR-TKI resistance. Clinical characteristics are shown in Table S5. 53.8% of EGFR-TKI-resistant specimens showed increased Mig-6 expression compared with matched baseline expression (14 of 26 cases). (Fig. 6a–f). These results support that Mig-6 is related to EGFR-TKI resistance in some contexts.

## Discussion

Although Mig-6 basically acts as an inhibitor of WT EGFR, the function of Mig-6 and interaction between EGFR and Mig-6 are completely different depending on the context including EGFR mutation, exposure to EGFR-TKI and phosphorylation of Mig-6.

Zhang et al. revealed that Mig-6 gene expression is differentially regulated in lung cancer and melanoma [21]. Maity et al. demonstrated that although Mig-6 deficiency reduces survival of mice due to accelerated tumorigenesis, mutant EGFRs can partially circumvent inhibition by Mig-6 in lung adenocarcinoma cell through phosphorylation of Mig-6 on key residue [22].

Most clinical data have shown that high Mig-6 expression is related to a poor prognosis of lung cancer [20, 23, 24]. Recently published study showed the different roles of Mig-6 in WT EGFR and mutant EGFR contexts [25]. Another study showed that hypoxia induces upregulation of Mig-6 which results in dormancy and resistance to EGFR-TKIs in primary cultured lung cancer cells with EGFR mutations [24].

We demonstrated that EGFR-TKI-resistant PC9/GR cells exhibit higher Mig-6 expression than EGFR-TKI-sensitive PC9 cells and that most of Mig-6 was phosphorylated. The combination of EGFR-TKI treatment and Mig-6 knockdown significantly overcame EGFR-TKI resistance and showed a significant synergistic effect in killing EGFR-TKI resistant PC9/GR cells. These data suggest that targeting Mig-6 can be the novel strategy to overcome EGFR-TKI resistance in lung cancer. Additionally, clinical data demonstrated that high Mig-6 expression is a predictor of poor prognosis in lung adenocarcinoma patients with and without EGFR mutation.

Xiaofei examined human head and neck squamous cell carcinoma and reported that acquired resistance to erlotinib was associated with the upregulation of Mig-6 and decreased EGFR activity [20]. However, this experiment does not reflect the actual clinical situation, because EGFR-TKIs are usually prescribed to patients with EGFR mutation, and most EGFR-mutated lung cancers are adenocarcinomas. Conversely, we selected PC9 cells, which harbor EGFR mutation, and PC9/GR cells, with an acquired EGFR T790M mutation, to mimic the real clinical setting.

In PC9/GR cells, the expression of EGFR was markedly decreased when Mig-6 was knocked down with shRNA. These data are consistent with the results that mutant EGFR phosphorylates Mig-6 and phosphorylated Mig6 decreases the degradation of EGFR mutants in lung adenocarcinoma [25].

Our data also showed that EGFR-TKI treatment increased the expression of Mig-6 in PC9/GR cells but not in PC9 cells (Fig. 4b). This result suggests that EGFR-TKI-resistant lung cancer cells dynamically react to EGFR-TKIs to survive under critical and specific contexts. To overcome EGFR-TKI resistance, one should target important mediators in this context. Our results suggest that upregulation and phosphorylation of Mig-6 is a key factor in cell survival and EGFR-TKI resistance. However, there may be some conditions of EGFR-TKI resistance in which Mig-6 is not changed or rather decreased. They may be caused by other significant mechanisms including small cell transformation, AXL activation and et al. [1, 2]. Therefore, selection of patients whose overexpression of Mig-6 is a critical cause of EGFR-TKI is clinically important and needs further study. In addition, we suggest targeting Mig-6 in two ways. One is inhibiting phosphorylation of Mig-6 by specific kinase inhibitor or dephosphorylating Mig-6 with phosphatase. And the other is inhibiting the effects of Mig-6 by targeting glycogen synthase kinase-3 (GSK-3), p70 ribosomal S6 kinase (S6K), or 1433 epsilon, because they were significantly altered when Mig-6 expression was increased, especially in patients with EGFR mutations in RPPA data (Table S6). It is known that inhibition of mTOR complex 1 activates GSK-3 beta pathway and EMT signaling [27], and GSK-3 promotes p70 ribosomal protein S6 kinase activity [28]. The mTOR inhibitor, which is conventionally used as a therapeutic agent in various solid cancers, including breast cancer and renal cell carcinoma, may serve as a substitute for the Mig-6 inhibitor.

We also confirmed the clinical aspects of Mig-6 by using our facility’s clinical samples and TCGA data. Consistent with our cell experiments, Mig-6 expressions of some cases were higher in re-biopsy samples acquired after obtaining EGFR-TKI resistance compared to initial biopsy samples. In these specific contexts with EGFR mutant and long EGFR-TKI exposure, phosphorylated Mig-6 does not function as a tumor suppressor, but rather contributes to the survival of cancer cells.

## Conclusion

**Fig. 7:**
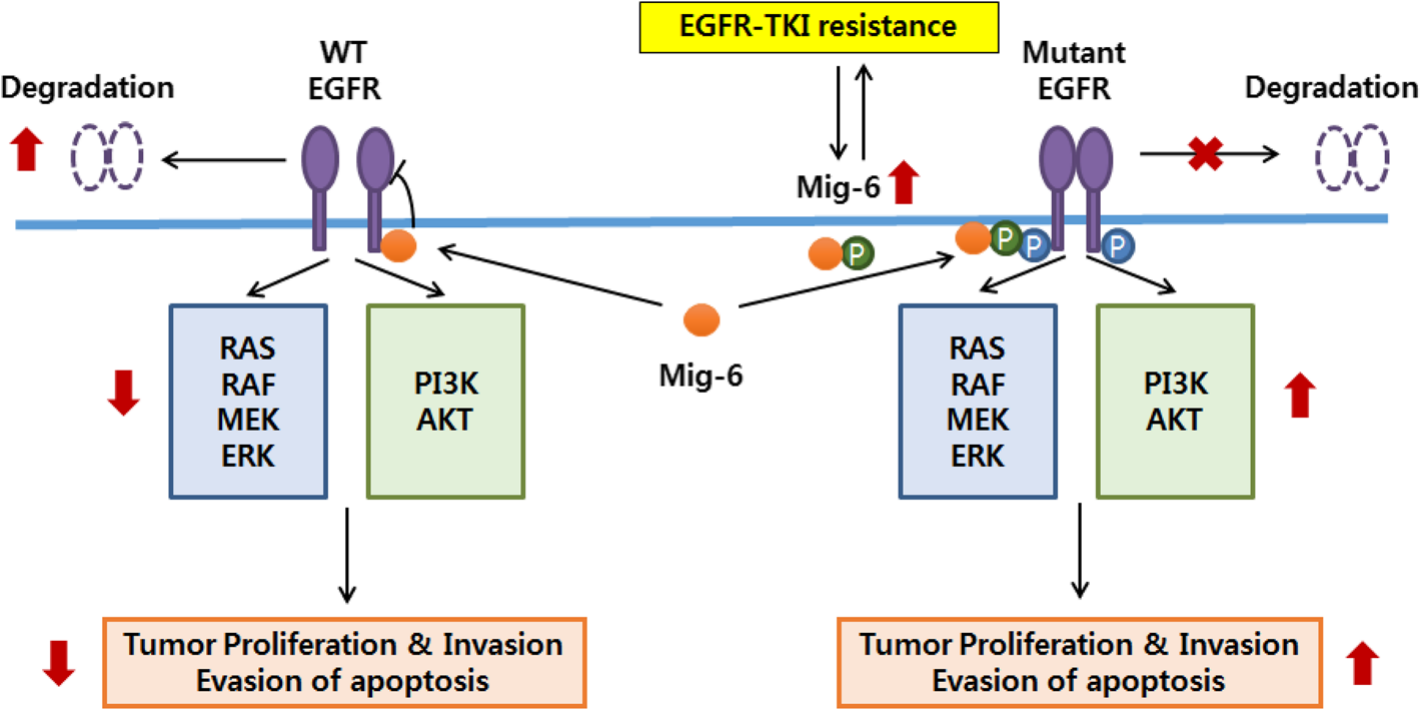
Graphical summary of Mig-6 function in EGFR-TKI resistant lung adenocarcinoma. Phosphorylated Mig-6 has a crucial oncogenic role in the survival of EGFR-mutant lung adenocarcinoma and targeting Mig-6 may be a promising strategy to overcome EGFR-TKI resistance in lung cancer

In summary, our data identified a crucial oncogenic role of Mig-6 in the survival of EGFR-TKI-resistant lung adenocarcinoma and we suggest targeting Mig-6 to overcome EGFR-TKI resistance in lung cancer (Fig. 7).

## Supplementary information

**Additional file 1.**

## Data Availability

All data generated or analyzed during this study are included in this article. Datasets used and/or analyzed during the current study are available from the corresponding author upon reasonable request.

## Abbreviations

EGFR: Epidermal growth factor receptor
TKI: Tyrosine kinase inhibitor
IHC: Immunohistochemistry
NSCLC: Non-small cell lung cancer
OS: Overall survival
TCGA: The Cancer Genome Atlas
