# Supplementary material for: Suppression of Mig-6 overcomes the acquired EGFR-TKI resistance of lung adenocarcinoma
Source: BMC Cancer. 2020 Jun 18;20:571. doi: 10.1186/s12885-020-07057-z (PMC7302243; doi:10.1186/s12885-020-07057-z)
Supplement: Supplementary file 1 — Additional file 1. [file 12885_2020_7057_MOESM1_ESM.pdf]

Table 1. Clinicopathologic parameters of patients from TCGA database (N=514)

Variables		Mean ± SD or number of patients (%)
Age, years		65.4 ± 9.9
Gender	Male	238 (46.3)
	Female	276 (53.7)
Smoking history	Never	165 (32.1)
	Former or current smoker	349 (67.9)
Anatomic site	RUL	186 (36.2)
	RML	21 (4.1)
	RLL	95 (18.5)
	LUL	120 (23.3)
	LLL	77 (15.0)
	Unknown	15 (2.9)
T stage	T1	170 (33.1)
	T2	279 (54.3)
	T3	46 (8.9)
	T4	19 (3.7)
Lymph node metastasis	N0	342 (66.5)
	N1	96 (18.7)
	N2	74 (14.4)
	N3	2 (0.4)
Metastasis	M0	489 (95.1)
	M1	25 (4.9)
EGFR mutation	Wild type	452 (87.9)
	Mutant	62 (12.1)

Table 2. Relationships between *ERRFI1* expression and clinicopathological factors in total patients (N=514)

Variables		No. of patients	*ERRFI1*		
			Low expression (N=257)	High expression (N=257)	*P* value
Age, years			66.0 ± 9.6	64.8 ± 10.2	0.182
Gender	Male	238 (46.3)	120 (46.7)	118 (45.9)	0.930
	Female	276 (53.7)	137 (53.3)	139 (54.1)	
Smoking history	Never	165 (32.1)	90 (35.0)	75 (29.2)	0.186
	Former or current	349 (67.9)	167 (65.0)	182 (70.8)	
Anatomic site	RUL	186 (36.2)	105 (40.9)	81 (31.5)	0.095
	RML	21 (4.1)	10 (3.9)	11 (4.3)	
	RLL	95 (18.5)	41 (16.0)	54 (21.0)	
	LUL	120 (23.3)	50 (19.5)	70 (27.2)	
	LLL	77 (15.0)	43 (16.7)	34 (13.2)	
	Unknown	15 (2.9)	8 (3.1)	7 (2.7)	
T stage	T1	170 (33.1)	102 (39.7)	68 (26.5)	0.009*
	T2	279 (54.3)	129 (50.2)	150 (58.4)	
	T3	46 (8.9)	17 (6.6)	29 (11.3)	
	T4	19 (3.7)	9 (3.5)	10 (3.9)	
Lymph node metastasis	N0	342 (66.5)	187 (72.8)	155 (60.3)	0.029*
	N1	96 (18.7)	39 (15.2)	57 (22.2)	
	N2	74 (14.4)	30 (11.7)	44 (17.1)	
	N3	2 (0.4)	1 (0.4)	1 (0.4)	
Metastasis	M0	489 (95.1)	244 (94.9)	245 (95.3)	1.000
	M1	25 (4.9)	13 (5.1)	12 (4.7)	
EGFR mutation	Wild type	452 (87.9)	221 (86.0)	231 (89.9)	0.223
	Mutant	62 (12.1)	36 (14.0)	26 (10.1)	

**P*< 0.05 between the two categories for a given variable.

Table 3. Upregulated signaling in group with high *ERRFI1* expression compared to in group with low *ERRFI1* expression using GO analysis (Total patients)

GO terms	Fold	*P value*
G1/S transition of mitotic cell cycle	2.99	0.015
Regulation of mitotic nuclear division	2.56	0.001
Mitotic cell cycle	2.31	<0.001
Response to hypoxia	2.27	0.002
Positive regulation of cell migration	2.12	<0.001
Angiogenesis	2.08	0.032
Positive regulation of cell proliferation	1.75	<0.001
Cellular response to cytokine stimulus	1.70	<0.001
Positive regulation of cell differentiation	1.62	0.006

Table 4. Upregulated signaling in group with high *ERRFI1* expression compared to in group with low *ERRFI1* expression using GO analysis (Patients with EGFR mutation)

GO terms	Fold	*P value*
Canonical glycolysis	8.07	0.029
Positive regulation of angiogenesis	3.22	0.009
Cellular response to tumor necrosis factor	2.84	0.003
Positive regulation of cell migration	2.45	<0.001
Positive regulation of cytokine production	2.24	0.023
Inflammatory response	2.21	0.007
Cytokine-mediated signaling pathway	1.99	0.017
Positive regulation of cell proliferation	1.90	<0.001
Positive regulation of cell differentiation	1.76	0.020

Table 5. Characteristics of patents who underwent re-biopsy of adenocarcinoma.

Patient	Age ranges	Initial stage	Initial biopsy (EGFR mutation)	Re-biopsy (acquired T790M)	Duration of 1st EGFR-TKI (months)	IHC
1	50's	IVB	E19 del	(+)	18	NA
2	60's	IVA	E19 del	(-)	17	CK7/CK19/TTF-1(+), ALK (-)
3	50's	IVB	L858R	(+)	10	TTF-1 (+), ALK (-)
4	70's	IVB	L858R	(-)	10	TTF-1/Napsin-A(+), CD56/p63(-), Synaptophysin(-) Ki-67 10%, ALK (-)
5	60's	IVA	L858R or L861Q	(-)	9	ALK (-)
6	50's	IVB	L858R or L861Q	(+)	26	ALK (-)
7	70's	IVB	L858R	(-)	5	NA
8	60's	IVB	E19 del	(+)	25	ALK (-)
9	50's	IVB	E19 del	(+)	18	ALK (-)
10	70's	IVB	G719X	(+)	18	ALK (-)
11	50's	IVA	E19 del	(+)	23	NA
12	60's	IVA	E19 del	(+)	10	ALK (-)
13	50's	IVB	L858R	(-)	16	NA
14	80's	IVA	G719X	(-)	16	TTF-1/Napsin-A(+), CD5/6(+), Synaptophysin(+) Ki-67 10%, ALK (-)
15	50's	IVB	L858R	(-)	5	TTF-1/Napsin-A(+), CD56/p63(-), Synaptophysin(+) Ki-67 20%, ALK (-)
16	60's	IVB	E19 del	(-)	42	TTF-1/Napsin-A(+), CD56/p63(-), Synaptophysin(-) Ki-67 10%
17	70's	IVA	E19 del	(-)	11	NA
18	50's	IVA	E19 del	(+)	7	TTF-1/Napsin-A(+), CD56/p63(-), Synaptophysin(-) Ki-67 10%, ALK (-)
19	50's	IV	L858R	(+)	16	CK7(+), CK20(+), TTF-1(+), CDX-2 (-), ALK (-)
20	30's	IV	19del	(-)	7	TTF-1 (+), ALK (-)
21	60's	IIIB	19del	(+)	10	ALK (-)
22	50's	IB	19del	(+)	14	CK7 (+), CK20 (-), TTF-1 (+/-)
23	80's	IVB	L858R or L861Q	(+)	16	TTF-1(+), ALK (-)
24	60's	IVB	19del	(+)	12	TTF-1/Napsin-A(+), CD56/p63(-), Synaptophysin(-) Ki-67 10%, ALK (-)
25	80's	IVA	19del	(+)	27	TTF-1/Napsin-A(+), CD56/p63(-), Synaptophysin(-) Ki-67 10%, ALK (-)
26	80's	IVA	19del	(-)	20	TTF-1/Napsin-A(+), CD56/p63(-), Synaptophysin(-) Ki-67 5%, ALK (-)

(NA : not analyzed)

Table 6. Comparison of variable proteins between decreased Mig6 expression group and increased Mig6 expression group in EGFR wild type and mutant patients using RPPA data

Variables	EGFR WT (N=214)				EGFR Mutant (N=23)			
	Mig6 expression change				Mig6 expression change			
	Negative (n=89)	Positive (n=125)	Fold change	*P* value	Negative (N=10)	Positive (N=13)	Fold change	*P* value
GSK3αβ	-0.30885	-0.2748	0.034052	0.324	-0.14767	-0.30333	-0.15566	0.019*
S6PS240S244	0.065217	-0.17288	-0.2381	0.001*	0.199263	-0.2597	-0.45896	0.020*
1433EPSILON	-0.17225	-0.12605	0.046195	0.060	-0.25718	-0.13258	0.124596	0.034*
AKTPT308	-0.35558	-0.53536	-0.17979	0.002*	-0.48504	-0.46223	0.022811	0.902
AKTPS473	-0.5156	-0.72743	-0.21183	0.011*	-0.31145	-0.29824	0.013214	0.961
CMETPY1235	0.042103	0.055544	0.013441	0.622	-0.06565	0.066223	0.131873	0.278
NCADHERIN	0.260356	0.116585	-0.14377	0.043*	-0.18846	-0.09498	0.093897	0.538
EGFRPY1068	1.137038	1.043156	-0.09388	0.623	3.654585	3.465294	-0.18929	0.842
P53	-0.62962	-0.51712	0.112502	0.081	-0.6666	-0.44694	0.219658	0.110

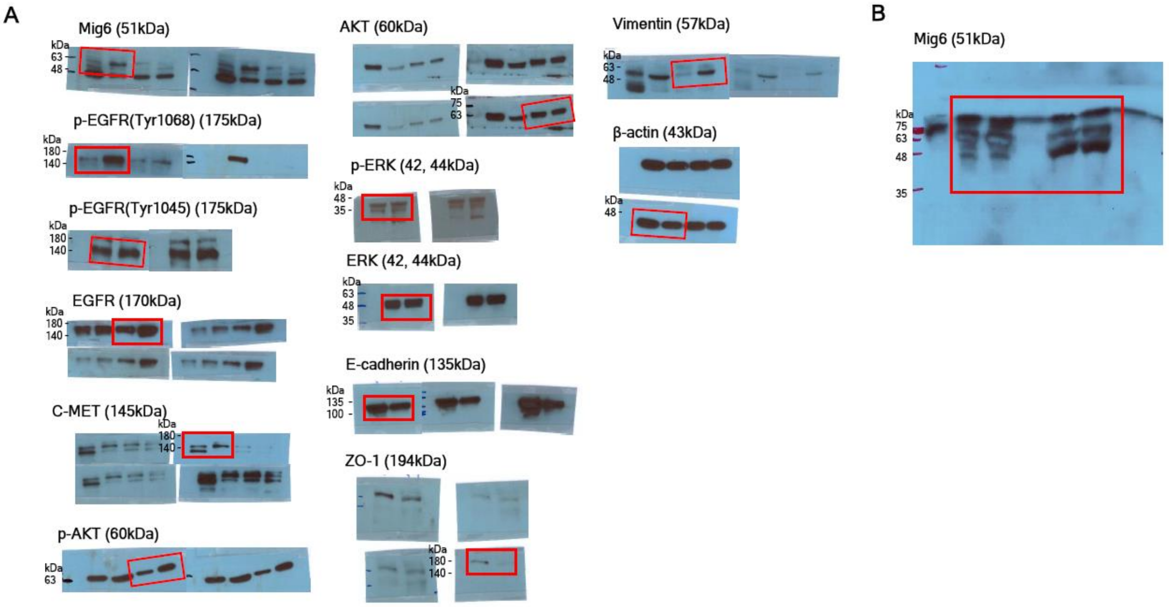

Fig. S1. (A) Original immunoblots of Mig-6, p-EGFR(Tyr1068), EGFR(Tyr1045), c-MET, p-AKT, AKT, p-ERK, ERT, E-cadherin, Zo-1, Vimentin and β-actin in **Fig. 1B**. (B) Original immunoblots of Mig-6 in **Fig. 1C**. Red rectangle indicates the cropped representative image in **Fig. 1.**

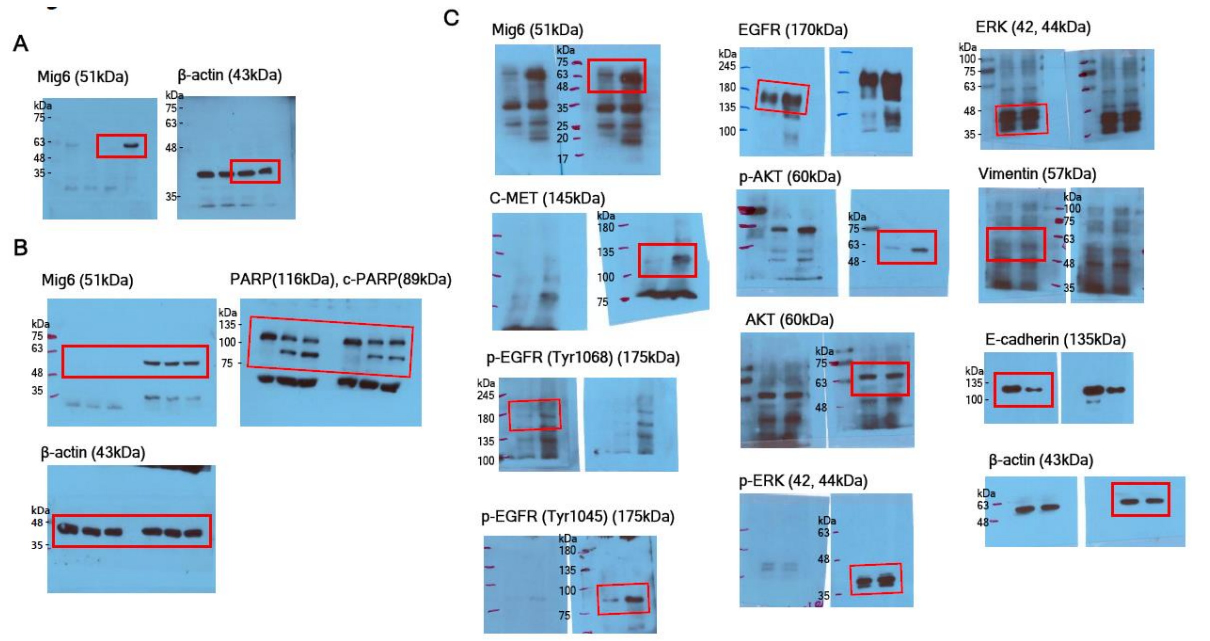

Fig. S2. (A) Original immunoblots of Mig-6 and β-actin in **Fig. 2A**. (B) Original immunoblots of Mig-6, PARP, c-PARP β-actin in **Fig. 2F**. (C) Original immunoblots of Mig-6, c-MET, p-EGFR(Tyr1066), p-EGFR(Tyr1045), EGFR, p-AKT, AKT, p-ERK, ERK, Vimentin, E-cadherin and β-actin in **Fig. 2G**. Red rectangle indicates the cropped representative image in **Fig. 2.**

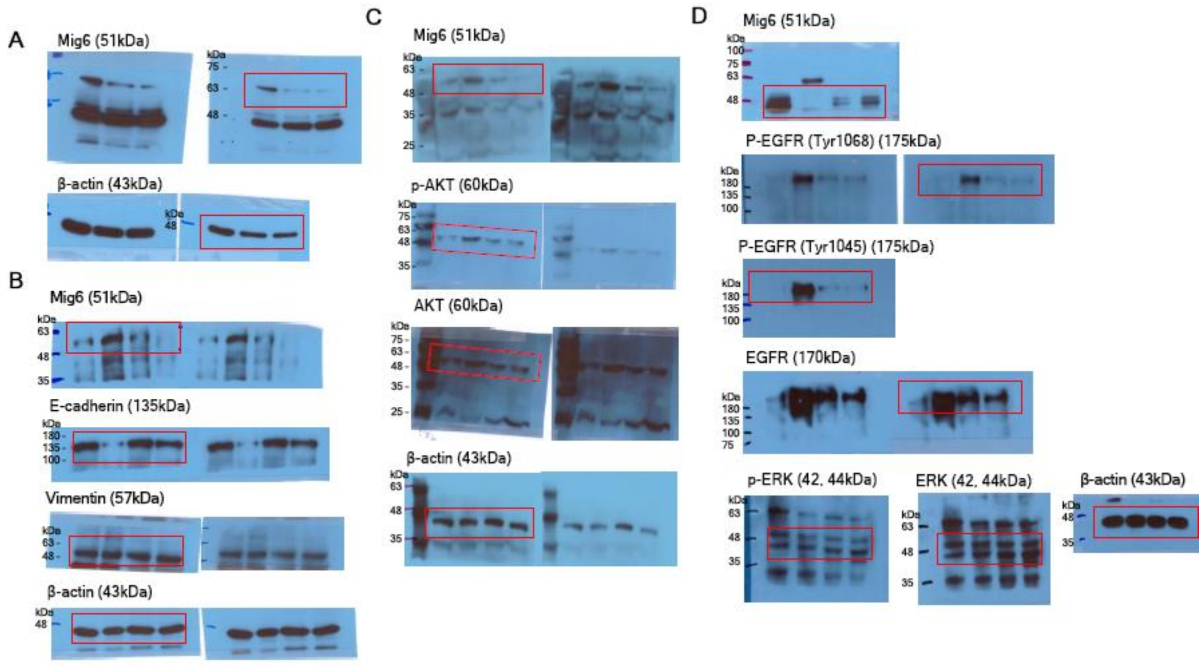

Fig. S3. (A) Original immunoblots of Mig-6 and β-actin in **Fig. 3A**. (B) Original immunoblots of Mig-6, E-cadherin, Vimentin, β-actin in **Fig. 3D.** (C) Original immunoblots of Mig-6, p-AKT, AKT, β-actin in **Fig. 3E**. (D) Original immunoblots of Mig-6, p-EGFR(Tyr1068), p-EGFR(Tyr1045), EGFR, p-ERK, ERK and β-actin in Fig. **3F.** Red rectangle indicates the cropped representative image in **Fig. 3.**

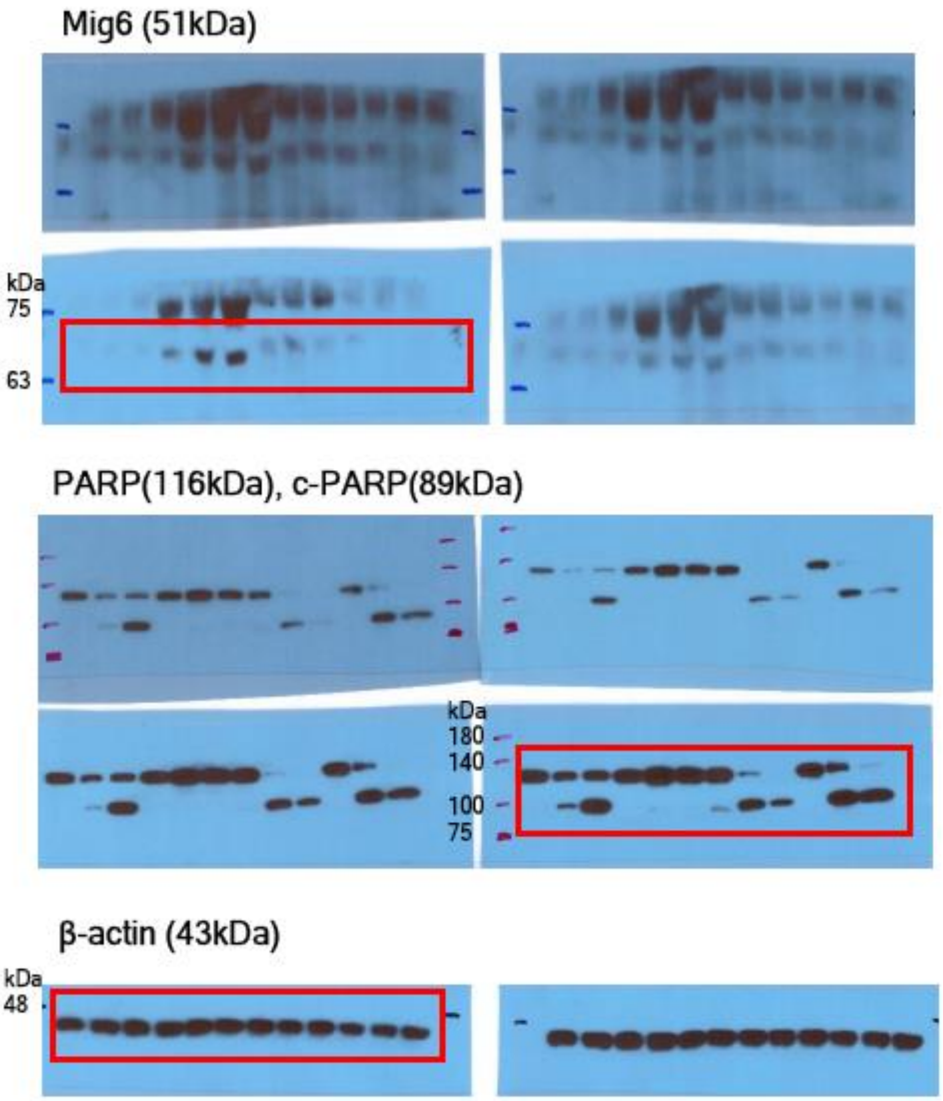

Fig. S4. Original immunoblots of Mig-6 PARP, c-PARP and β-actin in **Fig. 4B**. Red rectangle indicates the cropped representative image in **Fig. 4.**
